# *Astragalus membranaceus* ameliorates endometrial aging and restores receptivity by enhancing mitochondrial function in stromal and epithelial cells

**DOI:** 10.1093/procel/pwag012

**Published:** 2026-07-15

**Authors:** Xiunan Chen, Hongjuan Niu, Feng Deng, Yang Ke, Ziying Huang, Ke Ning, Zeyang Lin, Runan Pan, Jiayi Ma, Daiwen Xing, Yue Wang, Baoying Liao, Guangyao Lin, Yang Yu, Heng Pan, Ping Zhou, Rong Li

**Affiliations:** State Key Laboratory of Female Fertility Promotion, Center for Reproductive Medicine, Department of Obstetrics and Gynecology, Peking University Third Hospital, Beijing 100191, China; Key Laboratory of Assisted Reproduction, Peking University, Ministry of Education, Beijing 100191, China; Beijing Key Laboratory of Collaborative Innovation in Frontier Technologies for Population Quality, Beijing 100191, China; National Clinical Research Center for Obstetrics and Gynecology, Peking University Third Hospital, Beijing 100191, China; National Clinical Key Specialty Construction Program, Beijing 100191,China; State Key Laboratory of Female Fertility Promotion, Center for Reproductive Medicine, Department of Obstetrics and Gynecology, Peking University Third Hospital, Beijing 100191, China; Key Laboratory of Assisted Reproduction, Peking University, Ministry of Education, Beijing 100191, China; Beijing Key Laboratory of Collaborative Innovation in Frontier Technologies for Population Quality, Beijing 100191, China; National Clinical Research Center for Obstetrics and Gynecology, Peking University Third Hospital, Beijing 100191, China; National Clinical Key Specialty Construction Program, Beijing 100191,China; State Key Laboratory of Female Fertility Promotion, Center for Reproductive Medicine, Department of Obstetrics and Gynecology, Peking University Third Hospital, Beijing 100191, China; Key Laboratory of Assisted Reproduction, Peking University, Ministry of Education, Beijing 100191, China; Beijing Key Laboratory of Collaborative Innovation in Frontier Technologies for Population Quality, Beijing 100191, China; National Clinical Research Center for Obstetrics and Gynecology, Peking University Third Hospital, Beijing 100191, China; National Clinical Key Specialty Construction Program, Beijing 100191,China; Zhejiang Provincial Key Laboratory of Biometrology and Inspection & Quarantine, College of Life Sciences, Jiliang University, Hangzhou 310018, China; State Key Laboratory of Female Fertility Promotion, Center for Reproductive Medicine, Department of Obstetrics and Gynecology, Peking University Third Hospital, Beijing 100191, China; Key Laboratory of Assisted Reproduction, Peking University, Ministry of Education, Beijing 100191, China; Beijing Key Laboratory of Collaborative Innovation in Frontier Technologies for Population Quality, Beijing 100191, China; National Clinical Research Center for Obstetrics and Gynecology, Peking University Third Hospital, Beijing 100191, China; National Clinical Key Specialty Construction Program, Beijing 100191,China; State Key Laboratory of Female Fertility Promotion, Center for Reproductive Medicine, Department of Obstetrics and Gynecology, Peking University Third Hospital, Beijing 100191, China; Key Laboratory of Assisted Reproduction, Peking University, Ministry of Education, Beijing 100191, China; Beijing Key Laboratory of Collaborative Innovation in Frontier Technologies for Population Quality, Beijing 100191, China; National Clinical Research Center for Obstetrics and Gynecology, Peking University Third Hospital, Beijing 100191, China; National Clinical Key Specialty Construction Program, Beijing 100191,China; State Key Laboratory of Female Fertility Promotion, Center for Reproductive Medicine, Department of Obstetrics and Gynecology, Peking University Third Hospital, Beijing 100191, China; Key Laboratory of Assisted Reproduction, Peking University, Ministry of Education, Beijing 100191, China; Beijing Key Laboratory of Collaborative Innovation in Frontier Technologies for Population Quality, Beijing 100191, China; National Clinical Research Center for Obstetrics and Gynecology, Peking University Third Hospital, Beijing 100191, China; National Clinical Key Specialty Construction Program, Beijing 100191,China; State Key Laboratory of Female Fertility Promotion, Center for Reproductive Medicine, Department of Obstetrics and Gynecology, Peking University Third Hospital, Beijing 100191, China; Key Laboratory of Assisted Reproduction, Peking University, Ministry of Education, Beijing 100191, China; Beijing Key Laboratory of Collaborative Innovation in Frontier Technologies for Population Quality, Beijing 100191, China; National Clinical Research Center for Obstetrics and Gynecology, Peking University Third Hospital, Beijing 100191, China; National Clinical Key Specialty Construction Program, Beijing 100191,China; State Key Laboratory of Female Fertility Promotion, Center for Reproductive Medicine, Department of Obstetrics and Gynecology, Peking University Third Hospital, Beijing 100191, China; Key Laboratory of Assisted Reproduction, Peking University, Ministry of Education, Beijing 100191, China; Beijing Key Laboratory of Collaborative Innovation in Frontier Technologies for Population Quality, Beijing 100191, China; National Clinical Research Center for Obstetrics and Gynecology, Peking University Third Hospital, Beijing 100191, China; National Clinical Key Specialty Construction Program, Beijing 100191,China; State Key Laboratory of Female Fertility Promotion, Center for Reproductive Medicine, Department of Obstetrics and Gynecology, Peking University Third Hospital, Beijing 100191, China; Key Laboratory of Assisted Reproduction, Peking University, Ministry of Education, Beijing 100191, China; Beijing Key Laboratory of Collaborative Innovation in Frontier Technologies for Population Quality, Beijing 100191, China; National Clinical Research Center for Obstetrics and Gynecology, Peking University Third Hospital, Beijing 100191, China; National Clinical Key Specialty Construction Program, Beijing 100191,China; State Key Laboratory of Female Fertility Promotion, Center for Reproductive Medicine, Department of Obstetrics and Gynecology, Peking University Third Hospital, Beijing 100191, China; Key Laboratory of Assisted Reproduction, Peking University, Ministry of Education, Beijing 100191, China; Beijing Key Laboratory of Collaborative Innovation in Frontier Technologies for Population Quality, Beijing 100191, China; National Clinical Research Center for Obstetrics and Gynecology, Peking University Third Hospital, Beijing 100191, China; National Clinical Key Specialty Construction Program, Beijing 100191,China; State Key Laboratory of Female Fertility Promotion, Center for Reproductive Medicine, Department of Obstetrics and Gynecology, Peking University Third Hospital, Beijing 100191, China; Key Laboratory of Assisted Reproduction, Peking University, Ministry of Education, Beijing 100191, China; Beijing Key Laboratory of Collaborative Innovation in Frontier Technologies for Population Quality, Beijing 100191, China; National Clinical Research Center for Obstetrics and Gynecology, Peking University Third Hospital, Beijing 100191, China; National Clinical Key Specialty Construction Program, Beijing 100191,China; State Key Laboratory of Female Fertility Promotion, Center for Reproductive Medicine, Department of Obstetrics and Gynecology, Peking University Third Hospital, Beijing 100191, China; Key Laboratory of Assisted Reproduction, Peking University, Ministry of Education, Beijing 100191, China; Beijing Key Laboratory of Collaborative Innovation in Frontier Technologies for Population Quality, Beijing 100191, China; National Clinical Research Center for Obstetrics and Gynecology, Peking University Third Hospital, Beijing 100191, China; National Clinical Key Specialty Construction Program, Beijing 100191,China; State Key Laboratory of Female Fertility Promotion, Center for Reproductive Medicine, Department of Obstetrics and Gynecology, Peking University Third Hospital, Beijing 100191, China; Key Laboratory of Assisted Reproduction, Peking University, Ministry of Education, Beijing 100191, China; Beijing Key Laboratory of Collaborative Innovation in Frontier Technologies for Population Quality, Beijing 100191, China; National Clinical Research Center for Obstetrics and Gynecology, Peking University Third Hospital, Beijing 100191, China; National Clinical Key Specialty Construction Program, Beijing 100191,China; Clinical Stem Cell Research Center, Peking University Third Hospital, Beijing 100191, China; Beijing Advanced Center of Cellular Homeostasis and Aging-Related Diseases, Institute of Advanced Clinical Medicine, Peking University, Beijing 100191, China; Frontiers Medical Center, Tianfu Jincheng Laboratory, Chengdu 610200, China; Zhejiang Provincial Key Laboratory of Biometrology and Inspection & Quarantine, College of Life Sciences, Jiliang University, Hangzhou 310018, China; State Key Laboratory of Female Fertility Promotion, Center for Reproductive Medicine, Department of Obstetrics and Gynecology, Peking University Third Hospital, Beijing 100191, China; Key Laboratory of Assisted Reproduction, Peking University, Ministry of Education, Beijing 100191, China; Beijing Key Laboratory of Collaborative Innovation in Frontier Technologies for Population Quality, Beijing 100191, China; National Clinical Research Center for Obstetrics and Gynecology, Peking University Third Hospital, Beijing 100191, China; National Clinical Key Specialty Construction Program, Beijing 100191,China; State Key Laboratory of Female Fertility Promotion, Center for Reproductive Medicine, Department of Obstetrics and Gynecology, Peking University Third Hospital, Beijing 100191, China; Key Laboratory of Assisted Reproduction, Peking University, Ministry of Education, Beijing 100191, China; Beijing Key Laboratory of Collaborative Innovation in Frontier Technologies for Population Quality, Beijing 100191, China; National Clinical Research Center for Obstetrics and Gynecology, Peking University Third Hospital, Beijing 100191, China; National Clinical Key Specialty Construction Program, Beijing 100191,China; State Key Laboratory of Female Fertility Promotion, Center for Reproductive Medicine, Department of Obstetrics and Gynecology, Peking University Third Hospital, Beijing 100191, China; Key Laboratory of Assisted Reproduction, Peking University, Ministry of Education, Beijing 100191, China; Beijing Key Laboratory of Collaborative Innovation in Frontier Technologies for Population Quality, Beijing 100191, China; National Clinical Research Center for Obstetrics and Gynecology, Peking University Third Hospital, Beijing 100191, China; National Clinical Key Specialty Construction Program, Beijing 100191,China

**Dear Editor,** 

With the global trend toward delayed childbearing, pregnancy at advanced maternal age (>35 years) has become a major clinical challenge. Although poor reproductive outcomes in older women have traditionally been attributed primarily to age-related ovarian decline, increasing evidence indicates that the endometrium plays a critical and independent role in implantation failure and pregnancy loss in this population—an aspect that has long been underestimated (Pathare et al., 2023; Vitagliano et al., 2023; Zhao et al., 2023). Notably, even when euploid embryos are transferred, women of advanced maternal age exhibit reduced biochemical pregnancy, clinical pregnancy, and live birth rates compared with younger counterparts (Wang et al., 2025), underscoring the pivotal contribution of impaired endometrial receptivity to age-related fertility decline.

Age-associated endometrial dysfunction is increasingly recognized as a multifactorial process involving cellular senescence, mitochondrial impairment, and exacerbated oxidative stress (Lu et al., 2022; Chemerinski et al., 2024). Excessive accumulation of reactive oxygen species (ROS) disrupts cellular homeostasis, promotes DNA damage and cell-cycle arrest, and contributes to the development of a senescence-associated secretory phenotype, thereby creating a microenvironment that is unfavorable for embryo implantation (Palomba et al., 2021; Sharma, 2022). Accordingly, therapeutic strategies aimed at alleviating oxidative stress and preserving mitochondrial function may represent promising approaches to mitigate age-related endometrial decline.

*Astragalus membranaceus* (AM) is a widely used traditional medicinal herb that has been reported to exert antioxidant, anti-inflammatory, and cytoprotective effects in multiple aging-related contexts (Borowicz and Jach, 2025; Ma et al., 2025). These properties suggest that AM may contribute to the maintenance of tissue homeostasis under conditions of aging-driven stress. However, whether AM can modulate endometrial function and thereby support embryo implantation in the setting of advanced maternal age has not been systematically investigated.

Here, we report that AM treatment alleviates aging-linked endometrial senescence and partially restores uterine receptivity in aged mice. Using a combination of *in vivo* aging models, *in vitro* senescence systems, and supportive network pharmacology analyses, our findings suggest that the protective effects of AM are associated with reduced oxidative stress and preservation of mitochondrial function. Together, these observations support a potential role for AM as a non-hormonal intervention for mitigating age-related endometrial dysfunction.

In an established aged mouse model, we first examined whether AM treatment could alleviate age-associated endometrial senescence *in vivo*, while also performing a preliminary assessment of uterine safety (Fig. S1). Compared with young controls, aged uteri exhibited a pronounced increase in senescence-related markers, consistent with an accelerated senescent phenotype. Quantitative RT-PCR analysis showed that the mRNA levels of *Cdkn2a*, *Cdkn1a*, and *Tp53* were significantly elevated in the Aging group, whereas AM treatment markedly attenuated their expression (Fig. 1A). Consistently, Western blot analysis revealed increased P21 and P53 protein abundance in aged uteri, which was partially reversed following AM administration (Fig. 1B and 1C).

**Figure 1.**
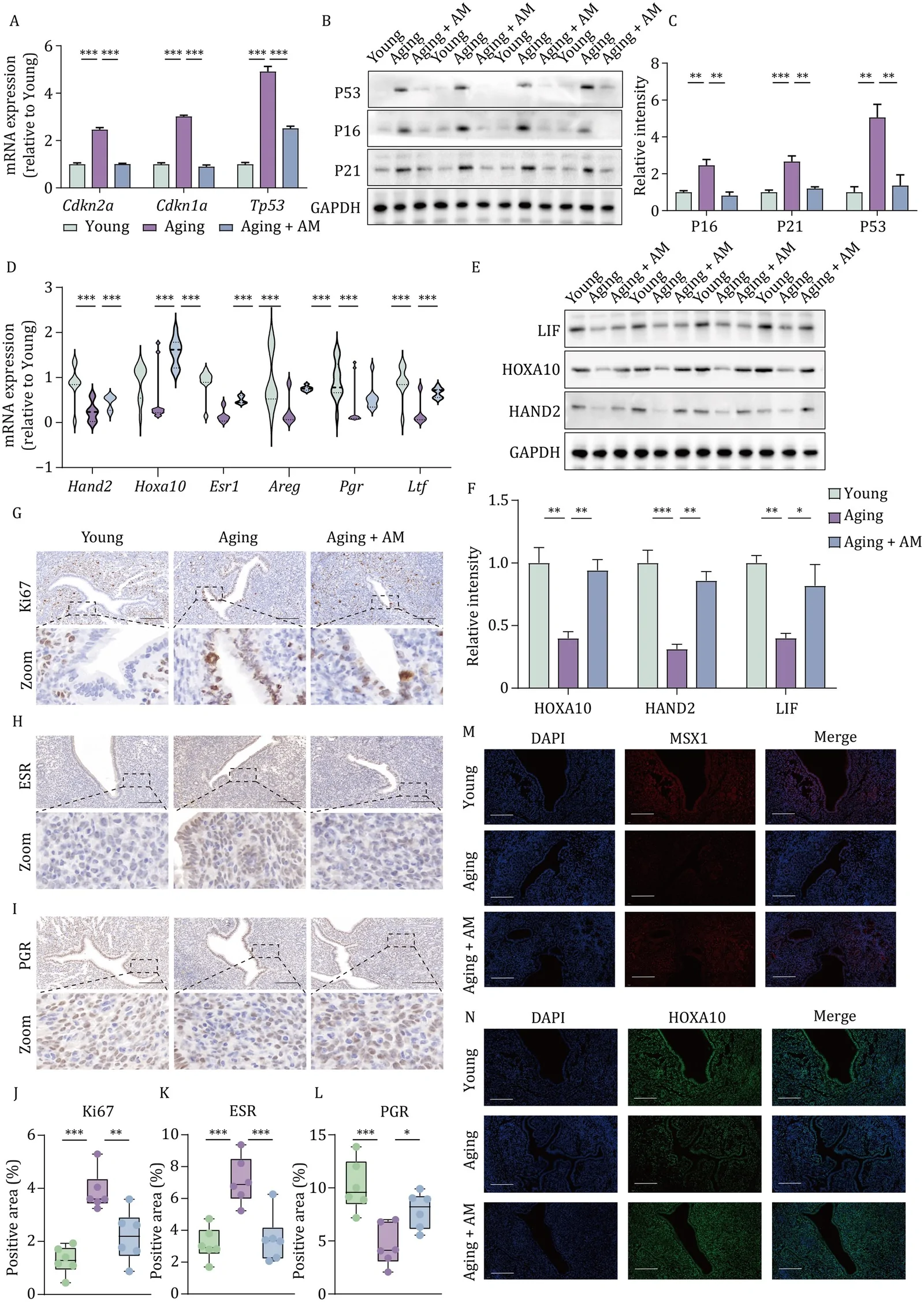
**AM alleviates uterine aging and restores endometrial receptivity-related phenotypes in aged mice**. (A) Heatmap showing regulon activity across groups, showing close similarity between young and QCT-treated samples and clear elevation in the aged group. (B) Representative Western blot images of P53, P16, and P21 protein expression in uterine tissues of different groups (GAPDH as endogenous control). (C) Quantitative analysis of the protein band intensities shown in (B), normalized to GAPDH. (D) Violin plots showing the relative mRNA levels of receptivity-related genes (*Hand2, Hoxa10, Esr1, Areg*, *Pgr*, and *Ltf*) across different groups (detected by qRT-PCR, normalized to *Gapdh*). (E) Representative Western blot images showing the expression of endometrial receptivity-related proteins LIF, HOXA10, and HAND2 (GAPDH as endogenous control). (F) Densitometric quantification of LIF, HOXA10, and HAND2 protein levels, presented as relative intensity to the Young group. (G–I) Representative immunohistochemical staining of Ki67, ESR, and PGR in uterine sections from Young, Aging, and AM + Aging groups, with corresponding higher-magnification images (ZOOM) (scale bar, 50 µm). (J–L) Quantitative analysis of the immunopositive areas for Ki67, estrogen receptor (ESR), and progesterone receptor (PGR) in uterine tissues. (M) Representative immunofluorescence images of MSX1 stainingwith DAPI nuclear counterstaining in uterine tissues (scale bar, 50 µm). (N) Representative immunofluorescence images of HOXA10 staining with DAPI nuclear counterstaining in uterine tissues (scale bar, 50 µm). Data are presented as mean ± SEM. Statistical significance was determined by one-way ANOVA with post hoc tests. *P < 0.05; **P < 0.01; ***P < 0.001. AM, *Astragalus membranaceus*; Aging, aged mouse group; Aging + AM, aged mouse group treated with AM; DAPI, 4′,6-diamidino-2-phenylindole; GAPDH, glyceraldehyde-3-phosphate dehydrogenase; qRT-PCR, quantitative real-time polymerase chain reaction; Young, young mouse group.

These molecular changes were accompanied by concordant histological and cellular alterations. Senescence-associated β-galactosidase (SA-β-gal) staining demonstrated a substantial accumulation of senescent cells in aged uterine tissues, which was markedly reduced after AM treatment (Fig. S2A). Immunohistochemical and immunofluorescence analyses further confirmed enhanced P21 and P53 signals in the endometrium of aged mice and their attenuation upon AM intervention (Figs. S2C–F, S3A, and S3B). Consistently, hematoxylin–eosin staining revealed disruption of endometrial architecture in aged mice, characterized by reduced glandular density and a loosely organized stromal compartment, whereas AM treatment partially restored endometrial integrity and tissue organization (Fig. S3E). Together, these findings indicate that AM treatment mitigates multiple molecular and histopathological features of age-related endometrial senescence *in vivo*.

To determine whether the alleviation of endometrial senescence was accompanied by functional improvement, we next examined markers associated with uterine receptivity. Compared with young controls, aged uteri exhibited a broad reduction in the expression of receptivity-related genes, including *Hand2*, *Hoxa10*, *Esr1*, *Areg*, *Pgr*, and *Ltf*, whereas AM treatment partially restored their mRNA levels (Fig. 1D). Consistently, Western blot analysis showed that the protein abundance of key receptivity regulators, including LIF, HOXA10, and HAND2, was markedly decreased in aged uteri and significantly increased following AM administration (Fig. 1E and 1F). Furthermore, immunohistochemical staining confirmed reduced HAND2 expression in the aged endometrium and its restoration after AM treatment (Fig. S3C, D). Immunofluorescence staining further showed decreased LIF expression and increased MUC1 expression in the aged endometrium, both of which were partially normalized following AM treatment (Fig. S4B, D, F). Concordantly, Ki67 staining revealed excessive epithelial-stromal proliferation in aged uteri, indicative of dysregulated endometrial dynamics, which was attenuated upon AM intervention (Fig. 1G and 1J). Moreover, aged endometria displayed an imbalance in steroid receptor expression, characterized by elevated ESR and reduced PGR levels; this aberrant pattern was partially corrected by AM treatment (Fig. 1H, 1I, 1K, and 1L). Immunofluorescence analyses of MSX1, MSX2, HOXA10, and COX2 further demonstrated that AM restored the expression levels of these key functional markers in aged endometrium (Fig. 1M, N; Fig. S4A, C, D). In summary, these results indicate that AM treatment not only mitigates endometrial senescence but also promotes functional recovery of uterine receptivity in aged mice.

To provide supportive mechanistic context for the observed effects of AM, we performed network pharmacology and bioinformatic analyses using a public transcriptomic dataset (GSE58144). Differential expression analysis identified 524 upregulated and 571 downregulated genes in aged endometrium (Fig. 2A). In parallel, putative targets corresponding to 22 bioactive compounds of AM were retrieved from public pharmacology databases (Table S1). Intersection of these targets with senescence-associated genes from the GSE58144 dataset yielded 55 overlapping genes (Fig. 2B), suggesting their potential relevance to AM-mediated regulation of endometrial aging. Functional enrichment analyses indicated that these overlapping targets were primarily associated with aging-related biological processes. KEGG pathway analysis highlighted cellular senescence-related signaling pathways, while GO enrichment analysis identified response to oxidative stress as a prominently enriched biological process (Fig. 2C and 2D). Detailed results from KEGG and GO enrichment analysis can be found in the Supplementary Materials (see “GO and KEGG enrichment analysis of intersection targets”). Consistent with these findings, protein–protein interaction analysis revealed several highly connected nodes, including *ESR1*, *HIF1A*, *CCNB1*, *KIT*, *MET*, and *PTGS2*, suggesting their potential roles within a broader regulatory network (Fig. S6). Collectively, these analyses support the notion that AM-associated targets converge on pathways related to oxidative stress regulation and senescence-related processes, providing complementary context for the experimental observations.

**Figure 2.**
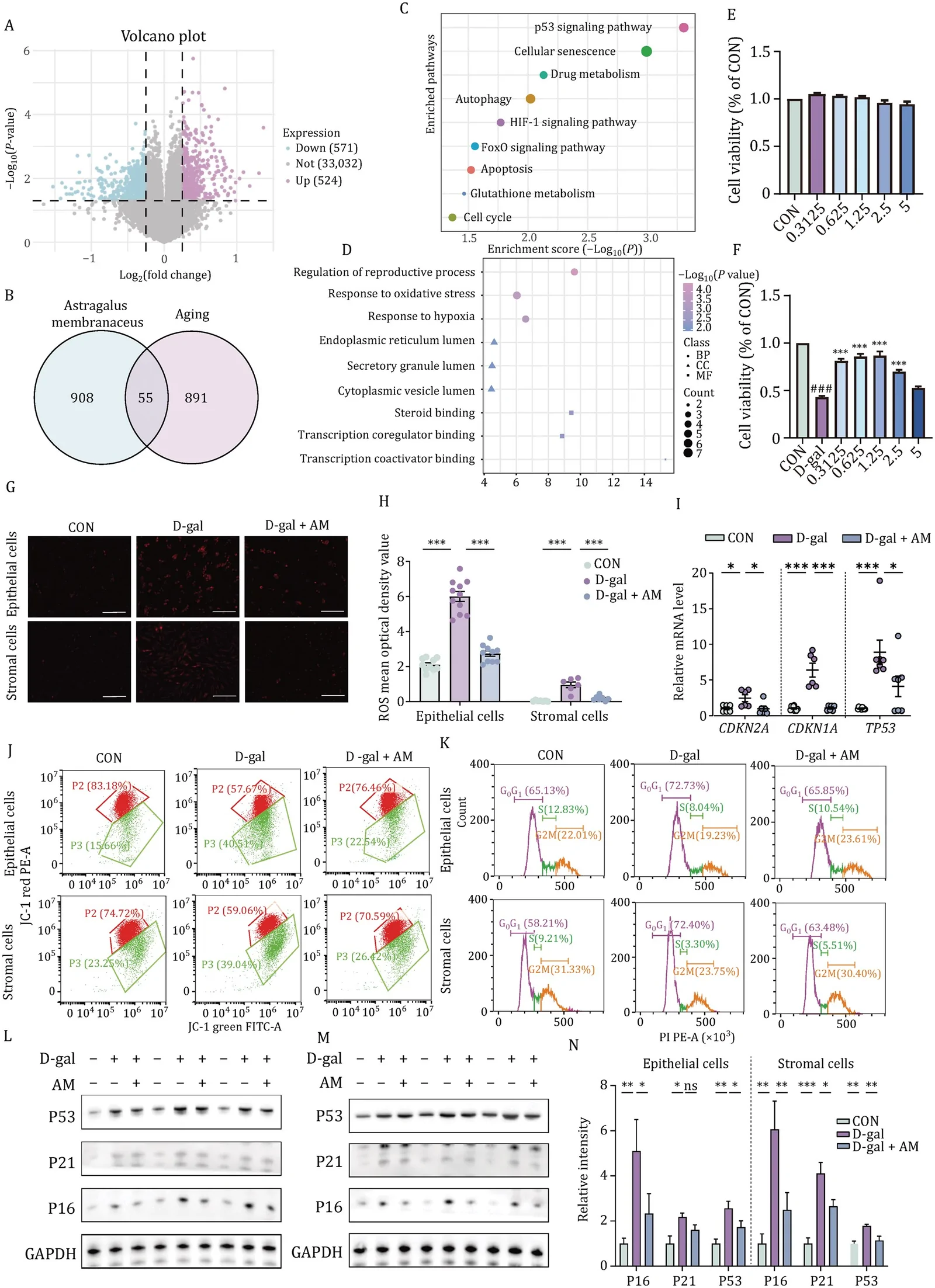
**AM ameliorates senescent phenotypes in endometrial stromal and epithelial cells**. (A) Volcano plot showing differentially expressed genes (DEGs) in aging vs. young endometrial tissues (Log_2_(fold change) >1 or <−1, adjusted *P *< 0.05). (B) Venn diagram displaying overlapping targets between AM-related targets and aging-related DEGs. (C) KEGG enrichment analysis of the overlapping targets, presenting the top enriched pathways. (D) Bubble plot showing the enriched GO terms of BP, CC, and MF. Node size represents the number of genes, and color intensity indicates the significance level (−Log_10_(*P* value)). (E) Cell viability of normal endometrial cells detected by CCK-8 assay (CON vs. CON + AM groups), aiming to evaluate the cytotoxicity of AM at experimental concentrations. (F) Cell viability of endometrial cells detected by CCK-8 assay (CON vs. D-gal vs. D-gal + AM groups), aiming to screen the optimal AM concentration for subsequent D-gal-induced senescence model intervention. (G) Representative ROS fluorescence staining images of endometrial epithelial and stromal cells in CON, D-gal, and D-gal + AM groups (scale bar, 50 µm). (H) Quantitative analysis of mean optical density (reflecting ROS levels) in endometrial epithelial and stromal cells among the three groups. (I) Relative mRNA levels of *CDKN2A*, *CDKN1A*, and *TP53* in endometrial epithelial and stromal cells (detected by qRT-PCR), with human *GAPDH* as the endogenous reference; gene expression was normalized to *GAPDH* and presented as fold change (2^−ΔΔCt^) relative to the CON group. (J) Mitochondrial membrane potential (detected by JC-1 staining and flow cytometry) in endometrial epithelial and stromal cells among the three groups, showing the proportion of cells with depolarized membrane potential (JC-1 Green^+^). (K) Cell cycle distribution (detected by flow cytometry) in endometrial epithelial and stromal cells among the three groups, displaying proportions of G_0_/G_1_, S, and G_2_/M phases. (L) (M) Western blot analysis of P53, P21, and P16 protein expressions in endometrial epithelial cells (L) and stromal cells (M), with GAPDH as the endogenous control. (N) Quantitative analysis of relative protein intensity (normalized to GAPDH) of P16, P21, and P53 in endometrial epithelial and stromal cells. Data were expressed as means ±SEM. *P < 0.05; **P < 0.01; ***P < 0.001. CON, control group; D-gal, D-galactose-induced senescence group; D-gal + AM, D-gal-induced group treated with *Astragalus membranaceus*; JC-1, 5,5′,6,6′-tetrachloro-1,1′,3,3′-tetraethylbenzimidazolylcarbocyanine iodide); KEGG, Kyoto Encyclopedia of Genes and Genomes; qRT-PCR, quantitative real-time polymerase chain reaction; ROS, reactive oxygen species.

To complement the *in vivo* findings, we established in vitro senescence models in endometrial stromal and epithelial cells using D-galactose (D-gal) treatment. Based on preliminary optimization, exposure to 40 mg/mL D-gal for 24 h was selected to induce cellular senescence (Fig. S5). CCK-8 assays showed that AM treatment at the tested concentrations did not significantly affect cell viability under basal conditions, indicating minimal cytotoxicity (Fig. 2E). In contrast, D-gal exposure markedly reduced cell viability, which was dose-dependently improved by AM treatment. Among the concentrations examined, 1.25 mg/mL AM exhibited the most pronounced protective effect and was therefore used in subsequent experiments (Fig. 2F). We next assessed oxidative stress and senescence-associated features *in vitro*. D-gal treatment led to a significant increase in intracellular ROS levels, whereas AM intervention markedly attenuated ROS accumulation (Fig. 2G and 2H). Consistent with these changes, D-gal markedly upregulated the expression of senescence-associated markers (*CDKN2A*, *CDKN1A*, and *TP53*), which were suppressed following AM treatment (Figs. 2I and S2B). In parallel, mitochondrial function analysis revealed a loss of mitochondrial membrane potential after D-gal exposure, as indicated by JC-1 staining, which was partially restored by AM treatment (Fig. 2J). Cell-cycle analysis further showed an increased proportion of cells arrested at the G_0_/G_1_ phase in the D-gal group, whereas AM intervention significantly normalized cell-cycle distribution (Fig. 2K). These regulatory effects were further supported by Western blot analysis at the protein level (Fig. 2L–N). Together, these *in vitro* observations are consistent with the *in vivo* findings and provide supportive evidence that AM alleviates oxidative stress-associated senescence phenotypes in endometrial cells. Notably, these protective effects were observed in both stromal and epithelial cells, indicating that AM exerts broadly comparable anti-senescent effects across distinct endometrial cell types under stress conditions.

In summary, this study demonstrates that AM intervention alleviates age-associated endometrial decline and promotes the restoration of uterine receptivity in aged mice. Given that impaired endometrial function represents a critical bottleneck in reproductive outcomes at advanced maternal age (Wang et al., 2025), our findings underscore the importance of targeting endometrial aging as a complementary strategy beyond conventional hormone-based approaches. We observed that AM treatment attenuated key senescence-associated features, including reduced SA-β-gal activity and downregulation of p53/p21 expression, while restoring the expression of essential receptivity-related markers and partially rebalancing ESR and PGR signaling. Together, these results suggest that AM may represent a potential non-hormonal approach for modulating age-related endometrial dysfunction, warranting further investigation in translational and clinical settings.

From a mechanistic perspective, our study integrates network pharmacology with experimental observations to provide supportive insight into pathways potentially involved in AM-mediated effects. Aging is widely recognized as a process characterized by the accumulation of molecular damage, which can be driven by mitochondrial dysfunction and excessive ROS production (Srivastava, 2017). In our models, AM treatment was associated with reduced ROS accumulation and partial restoration of mitochondrial membrane potential, accompanied by attenuation of p53/p21-associated senescence features in endometrial stromal cells. These experimentally validated alterations provided the primary rationale for focusing the discussion on oxidative stress–mitochondrial pathways, which were consistently supported across both *in vivo* aging models and *in vitro* senescence assays. These findings indicate that mitochondrial dysfunction and oxidative stress represent important cellular features accompanying aging-dependent endometrial senescence. Given the multifactorial nature of reproductive aging, mitochondrial alterations may act in concert with other aging-related pathways to shape the senescent endometrial phenotype, and their precise causal contribution remains to be further clarified through targeted interventions focusing on mitochondrial function. In this context, the comparable protective effects observed in both stromal and epithelial cells are more likely to reflect shared cellular responses to age-associated stress conditions rather than preferential targeting of a specific cell type. Given that both compartments are highly sensitive to oxidative stress and senescence-related signals, AM may act by modulating stress-adaptive processes that are commonly operative across different endometrial cell populations.

Although validation in human clinical samples will be required to account for species-specific differences, the present findings provide supportive experimental evidence for the potential relevance of AM in the context of aging-linked endometrial dysfunction. Importantly, the observed protective effects in this study are attributed to the standardized whole extract of AM, reflecting the multi-component nature of this herbal medicine. We acknowledge that the biological activity of AM is likely mediated through the coordinated actions of multiple bioactive constituents, which may represent both a therapeutic advantage and a challenge for reproducibility and clinical translation. Future studies will therefore focus on identifying key active components of AM and elucidating their molecular targets, with the aim of refining mechanistic understanding and facilitating translational development. In conclusion, this study provides a conceptual and experimental framework supporting the potential of AM as a non-hormonal strategy for modulating age-related endometrial dysfunction and reproductive aging.

## Footnotes

We thank the staff of the Reproductive Medicine Center, Department of Obstetrics and Gynecology, Peking University Third Hospital for their support.

This work was supported by the National Natural Science Foundation of China (82288102 and 82571918), the National Key Research and Development Project of China (2025YFC2708000), Beijing Natural Science Foundation (7252152), the Major and Difficult Diseases Clinical Collaboration Project of the National Administration of Traditional Chinese Medicine (ZDYN-2024-B-008, ZDYN-2024-A-068), Frontiers Medical Center, Tianfu Jincheng Laboratory Foundation (TFJCPI20250032). We would like to thank all the patients and staff of Reproductive Medicine Center, Department of Obstetrics and Gynecology, Peking University Third Hospital for their contributions.

The authors declare that they have no conflict of interest.

This study did not involve human tissue samples. All animal experiments were conducted in accordance with institutional guidelines for the care and use of laboratory animals and were approved by the Institutional Animal Care and Use Committee of Peking University Third Hospital (Approval No. A20250165).

Publicly available transcriptomic data were obtained from the Gene Expression Omnibus (GEO) under accession number GSE58144. Other data supporting the findings of this study are available from the corresponding author upon reasonable request.

Rong Li, Ping Zhou, Heng Pan, and Yang Yu conceived the study. Xiunan Chen, Hongjuan Niu, Feng Deng, and Yang Ke performed animal modeling, drug treatment, cell culture, network pharmacology analysis, qRT-PCR, Western blotting, SA-β-gal staining, and immunostaining, and analyzed the data. Ke Ning, Zeyang Lin, Runan Pan, Jiayi Ma, Daiwen Xing, Yue Wang, Baoying Liao, and Guangyao Lin provided technical assistance. Xiunan Chen wrote the manuscript. All authors reviewed and approved the final manuscript.

## Supplementary Material

pwag012_Supplementary_Data
