# Supplementary material for: Astragalus membranaceus ameliorates endometrial aging and restores receptivity by enhancing mitochondrial function in stromal and epithelial cells
Source: Protein Cell. 2026 Jul 15;17(9):876–82. doi: 10.1093/procel/pwag012 (PMC13521270; doi:10.1093/procel/pwag012)
Supplement: Supplementary file 1 [file pwag012_supplementary_data.zip › pwag012_Supplementary_Data/PAC-25869-LR-Supplementary material-.pdf]

I: Materials and Methods
II: Supplementary Figures and Figure legends

I: Materials and Methods

Animal feeding and treatments

8-week-old and 10-month-old mice(Wang et al., 2025) were obtained from the Animal Care and Treatment Committee of the Peking University Health Science Center. 10-month-old female mice were used to model age-associated reproductive decline rather than advanced systemic aging, as reproductive aging in mice precedes somatic aging. All animals were housed under standardized conditions with a 12-hour light/dark cycle, ambient temperature maintained at 20-25 °C, and relative humidity of 55% ± 10%. Food and water were provided ad libitum throughout the study period.

Cell culture, AM treatment, and senescence detection

The human endometrial epithelial cell line (Ishikawa) and human endometrial stromal cell line (HESCs) were cultured in Dulbecco's modified Eagle's medium/Ham's F12 (DMEM/F12; Gibco, USA) supplemented with 10% fetal bovine serum (FBS; Gibco, USA) and 1% penicillin-streptomycin (Gibco, USA). Cells were maintained at 37 °C in a humidified atmosphere containing 5% CO_2.

For treatment, Ishikawa cells and HESCs were exposed to 1.25 mg/mL AM for 12 hours, whereas cellular senescence in Ishikawa cells and HESCs was induced by D-galactose (D-gal; Solarbio, China). Senescent cells were identified using a Senescence β-Galactosidase Staining Kit (Cell Signaling Technology, USA) following the manufacturer's instructions. Each experiment was performed independently in triplicate.

Cell viability assay

Cell viability was evaluated using a Cell Counting Kit-8 (CCK-8; Apexbio, USA) following the manufacturer's protocol. Cells were seeded into 96-well plates at a density of 2.5×10^4 cells per well and allowed to adhere overnight. After overnight adhesion, they were treated with various concentrations of D-galactose (10, 20, 30, 40,

50, and 60 mg/ml) for 24 hours. Based on the CCK-8 results, the optimal concentration of D-galactose (40 mg/ml) for inducing senescence was selected for subsequent experiments. Then, to evaluate the protective effects of AM extract, the cellular senescence model was induced with the selected D-galactose concentration. Subsequently, the senescent cells were treated with a range of concentrations of AM extract (0, 0.3125, 0.625, 1.25, 2.5, and 5 mg/ml) for 24 hours, followed by the CCK-8 assay.

After treatment, 10 μL of CCK-8 reagent was added to each well, and the plates were incubated at 37 °C for 2 hours. The optical density (OD) values were measured at 450 nm using a microplate reader (Multiskan GO, Thermo Fisher Scientific, USA) after 60 seconds of gentle shaking. Cell viability was calculated as a percentage relative to the control group, which was defined as 100% viable cells. All experiments were performed in triplicate (n = 3).

Measurement of intracellular reactive oxygen species (ROS)

Intracellular reactive oxygen species (ROS) levels were determined using a commercial ROS assay kit (Elabscience, China; Cat. No. E-RC-F005) following the manufacturer’s instructions. Cells were seeded in 6-well plates and cultured until reaching approximately 70-80% confluence. To investigate the protective effects of AM extract against D-galactose-induced ROS generation, cells were divided into different groups and treated for 24 hours as follows: the control group (untreated), the model group (treated with 40 mg/ml D-galactose alone), and the intervention groups (co-treated with 40 mg/ml D-galactose and 1.25 mg/ml AM extract). After the treatments, cells were incubated with the ROS-specific red fluorescent probe at 37 °C for 30 minutes in the dark.

Following incubation, cells were gently washed three times with PBS to remove residual dye. The red fluorescence intensity, indicative of intracellular ROS accumulation, was observed under a fluorescence microscope (Zeiss Axio Vert. A1) using appropriate excitation/emission wavelengths. Quantitative fluorescence analysis was performed using ImageJ software. Results were expressed as the relative

fluorescence intensity compared with the control group, representing the cellular ROS level. All experiments were independently repeated three times (n = 3).

Mitochondrial membrane potential assays

Mitochondrial membrane potential was detected using a JC-1 staining kit (Beyotime, China; C2006) according to the manufacturer's instructions. Briefly, cells were collected, washed with PBS, and incubated with freshly prepared JC-1 working solution at 37 °C for 30 min in the dark. After incubation, cells were washed with 1× JC-1 buffer and resuspended in 200 μL buffer before acquisition. The red/green fluorescence ratio was used as an indicator of mitochondrial depolarization.

Cell cycle analysis by flow cytometry

Flow cytometric analyses were performed to assess mitochondrial membrane potential and cell cycle distribution across the three groups (control, senescence/model, and AM-treated). All measurements were carried out on a CytoFLEX S flow cytometer (Beckman Coulter, USA), and data were analyzed using CytExpert software version 2.0.

Cell cycle distribution was determined using a PI-based Cell Cycle Detection Kit (4A Biotech, China; FXP0211). In brief, cells were harvested, washed with PBS, and fixed in 75% pre-chilled ethanol at 4 °C for ≥2 h. Fixed cells were washed, incubated with staining solution containing PI and RNase A at 37 °C for 30 min in the dark, and subsequently analyzed by flow cytometry. The proportions of cells in G0/G1, S, and G2/M phases were calculated based on PI fluorescence histograms. All flow cytometry experiments were independently repeated three times.

Hematoxylin and eosin (H&E) staining, immunofluorescence and immunohistochemistry

For histological examination, endometrial tissues were fixed in 4% paraformaldehyde, dehydrated, and embedded in paraffin. Serial sections (5 μm thick) were prepared, deparaffinized, and rehydrated through graded ethanol series. The slides were then

stained with hematoxylin and eosin to evaluate general tissue morphology and cellular architecture.

For immunofluorescence analysis, cultured endometrial cells were fixed with 4% paraformaldehyde (PFA) for 15 minutes, permeabilized using 0.1% Triton X-100, and subsequently blocked in 1% bovine serum albumin (BSA) for 30 minutes at room temperature. The samples were incubated overnight at 4 °C with the following primary antibodies: MSX-2 (PA5-106670, Thermofisher, USA; 1:500), COX2 (ab168745, Abcam, UK; 1:500), ESR (ab259427, Abcam, UK; 1:500), LIF (PA5-115510, Thermfisher, USA; 1:500); P21 (MA5-31479, Thermfisher, USA; 1:500); MUC1 (MA5-11202, Thermfisher, USA; 1:500). After washing with PBS, slides were treated with the appropriate fluorophore-conjugated secondary antibodies for 1 hour at room temperature. Nuclei were counterstained with DAPI (1 μg/mL; Invitrogen, USA) for visualization. Fluorescent images were acquired using a Zeiss LSM 710 confocal laser scanning microscope (Carl Zeiss, Germany) under identical exposure settings across groups.

For immunohistochemistry, the uterine tissues were fixed in 4% PFA, embedded in paraffin, and sliced into 5 μm sections. After deparaffinization in xylene and rehydration via a graded ethanol sequence, antigen retrieval was carried out by heating the sections in sodium citrate buffer (pH 6.0) at 99°C for 15 minutes. Endogenous peroxidase activity was inhibited using 3% hydrogen peroxide, and non-specific binding sites were blocked with normal goat serum. The sections were subsequently incubated with primary antibodies specific to P21, P53, Ki67, HAND2, PGR, ESR, COL1A1, FN1, and ERα at 4°C overnight. Following washing, the sections were exposed to an HRP-conjugated secondary antibody (Polymer-HRP Detection System (Rabbit), Bioss, China) for 1 hour at room temperature. Signal detection was performed using a DAB substrate kit (Acmec, China), and the sections were counterstained with hematoxylin. Stained images were captured using a Primo Star light microscope (Carl Zeiss, Germany). For each staining run, negative controls were included by substituting the primary antibody with normal rabbit IgG.

Western blotting

Proteins were extracted from mouse uterine tissues, epithelial cells, and stromal cells by homogenization in T-PER™ lysis buffer (Thermo Scientific, USA) containing protease and phosphatase inhibitors. A total of 20 μg protein per lane was separated by SDS-PAGE and transferred to PVDF membranes. After blocking with 5% non-fat milk, the membranes were incubated overnight at 4°C with primary antibodies against the following proteins: P16, P21, P53, HOXA10, HAND2, LIF, and GAPDH, followed by incubation with appropriate specific secondary antibodies. Protein bands were visualized using an ECL substrate, and immunoreactive bands were quantified by densitometry using ImageJ software (NIH, USA).

qRT-PCR

Total RNA was isolated from mouse uterine epithelial and stromal cells utilizing TRIzol™ reagent (Invitrogen) following the manufacturer's protocols. RNA concentration and purity were assessed using a NanoDrop spectrophotometer. Complementary DNA (cDNA) was generated from 1μg of total RNA with the RevertAid First-Strand cDNA Synthesis Kit (Thermo Fisher Scientific). Quantitative real-time PCR was conducted on an ABI 7500 instrument (Applied Biosystems, NY) utilizing SYBR Green Realtime PCR Master Mix (Applied Biosystems, NY). The expression levels of key genes involved in uterine receptivity, and cell cycle regulation were analyzed. Primer sequences used were provided in Table S2. Target gene mRNA expression was normalized to the housekeeping gene GAPDH, and the relative expression fold changes were calculated via the $2^{-\Delta\Delta Ct}$ method.

Analysis of AM-related targets and DEGs in aging endometrium

This study utilized TCMSP, BATMAN, and ETCM database as screening tools to perform a preliminary compounds analysis of AM. After eliminating duplicate chemical compositions, potential chemical compositions were obtained based on the screening criteria of oral bioavailabolity (OB) ≥ 30% and drug-like (DL) ≥ 0.18. Then, the corresponding results were obtained and analyzed. The Swiss Target Prediction,

PharmMapper, TCMSP, and ETCM database were applied to identify component-related targets. Finally, the prediction results from these databases were integrated, duplicates were removed. GSE58144 dataset was retrieved to identify differentially expressed genes (DEGs) between aging endometrium (≥35 years) and young control tissues in mid-secretory phase. This dataset contained transcriptomic data from 43 aging tissue samples and 9 young tissues. DEGs were screened with the thresholds of $|\log2(\text{fold change})| \geq 0.25$ and P value <0.05. Visualization of the DEGs was conducted using the R4.4.0 software which generated a volcano plot.

GO and KEGG enrichment analysis of intersection targets

The intersection of AM-related targets and DEGs was determined using Venn for visualizing overlapping genes, as potential targets of AM in the treatment of aging endometrium. To investigate the biological functions and signaling pathways linked to the intersection targets between AM and aging endometrium, GO and KEGG enrichment analyses were conducted with R4.4.0 software. KEGG enrichment analysis revealed that the overlapping targets between AM-related compounds and senescence-associated genes were predominantly associated with aging-related pathways, including cellular senescence, p53 signaling, FoxO signaling, autophagy, and apoptosis. Finally, the results were visually represented for bubble charts using the R4.4.0 software.

Construction of PPI network and compound-target-pathway network

A protein-protein interaction (PPI) network on the potential targets associated with aging endometrium improved by AM were visualized and analyzed with Cytoscape 3.8.0. Network topology analysis with the Cytohubba plugin pinpointed hub targets by betweenness, degree, and closeness centrality. Based on the obtained chemical components, common targets and signaling pathways, a Sankey diagram of components-targets-signaling pathways was drawn using R4.4.0 software to further screen the main chemical components, targets and signaling pathways for subsequent in-depth research.

Statistical analysis and experimental reproducibility

The experiment data were presented as the mean ± SEM, with the sample size (n) for each group specified in figure legends. All experiments were repeated independently at least three times to ensure reproducibility, with consistent results across replicates. Statistical analyses were performed using GraphPad Prism software (Version 9; GraphPad Prism; San Diego, CA, USA) after verifying data normality (Shapiro-Wilk test) and variance homogeneity (Levene's test). Two independent groups were compared via two-tailed Student's t-test, while three or more groups were analyzed by one-way analysis of variance (one-way ANOVA). A P-value < 0.05 was considered statistically significant.

References

1. Wang, Y., Zhou, P., Shan, H., Liu, X., Cheng, M., Ye, Z., Chen, X., Liao, B., Peng, T., Xiao, C., *et al.* (2025). Endometrial aging is accompanied by H3K27ac and PGR loss. Nature Aging 5, 816-830.

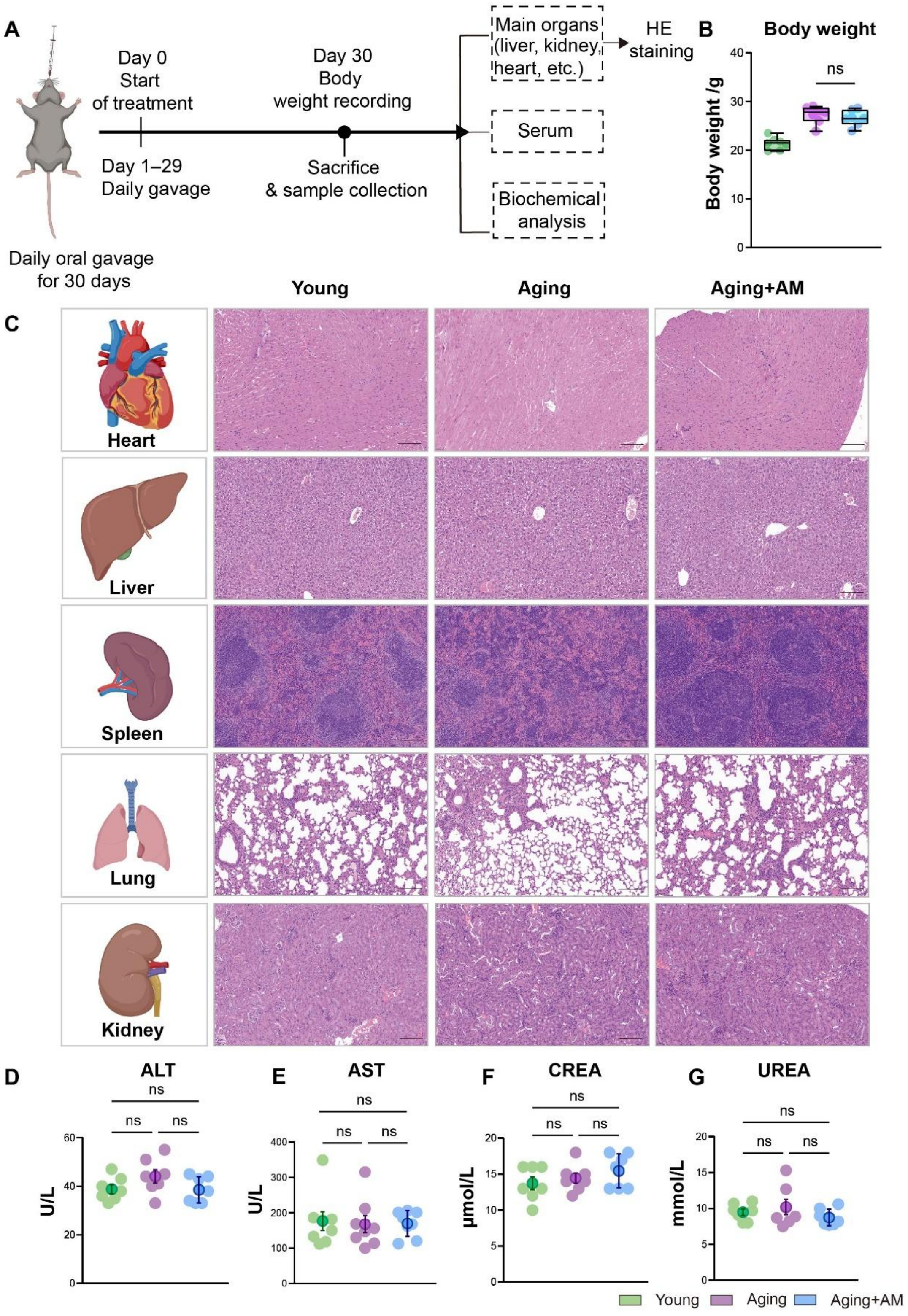
A
Day 0
Start
of treatment
Day 1–29
Daily gavage
Day 30
Body
weight recording
Sacrifice
& sample collection
Main organs
(liver, kidney,
heart, etc.)
HE
staining
Serum
Biochemical
analysis
Daily oral gavage
for 30 days
B
Body weight
Body weight /g
ns
C
Young
Aging
Aging+AM
Heart
Liver
Spleen
Lung
Kidney
D
ALT
U/L
ns
E
AST
U/L
F
CREA
μmol/L
G
UREA
mmol/L
Young
Aging
Aging+AM

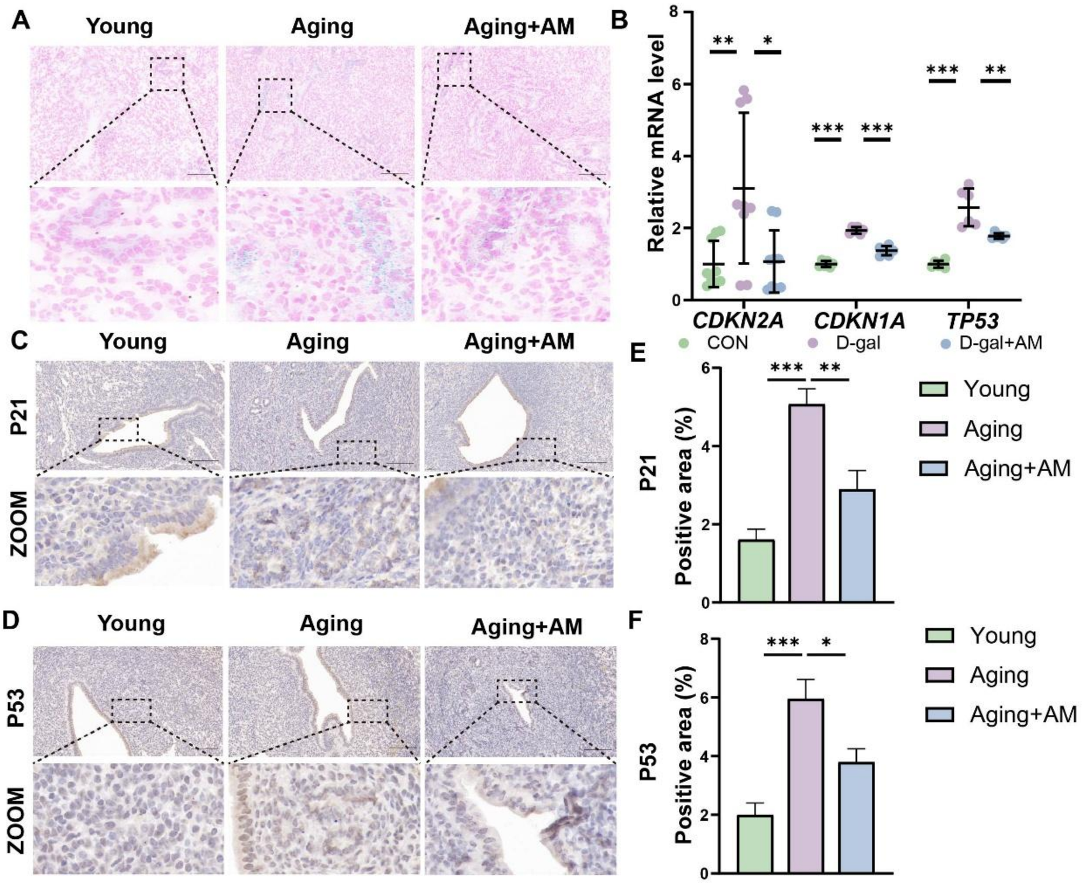

A
Young
Aging
Aging+AM
B
Relative mRNA level
8
6
4
2
0
**
*
***
***
***
**
CDKN2A
CDKN1A
TP53
CON
D-gal
D-gal+AM
C
Young
Aging
Aging+AM
P21
ZOOM
E
P21
Positive area (%)
6
4
2
0
***
**
Young
Aging
Aging+AM
D
Young
Aging
Aging+AM
P53
ZOOM
F
P53
Positive area (%)
8
6
4
2
0
***
*
Young
Aging
Aging+AM

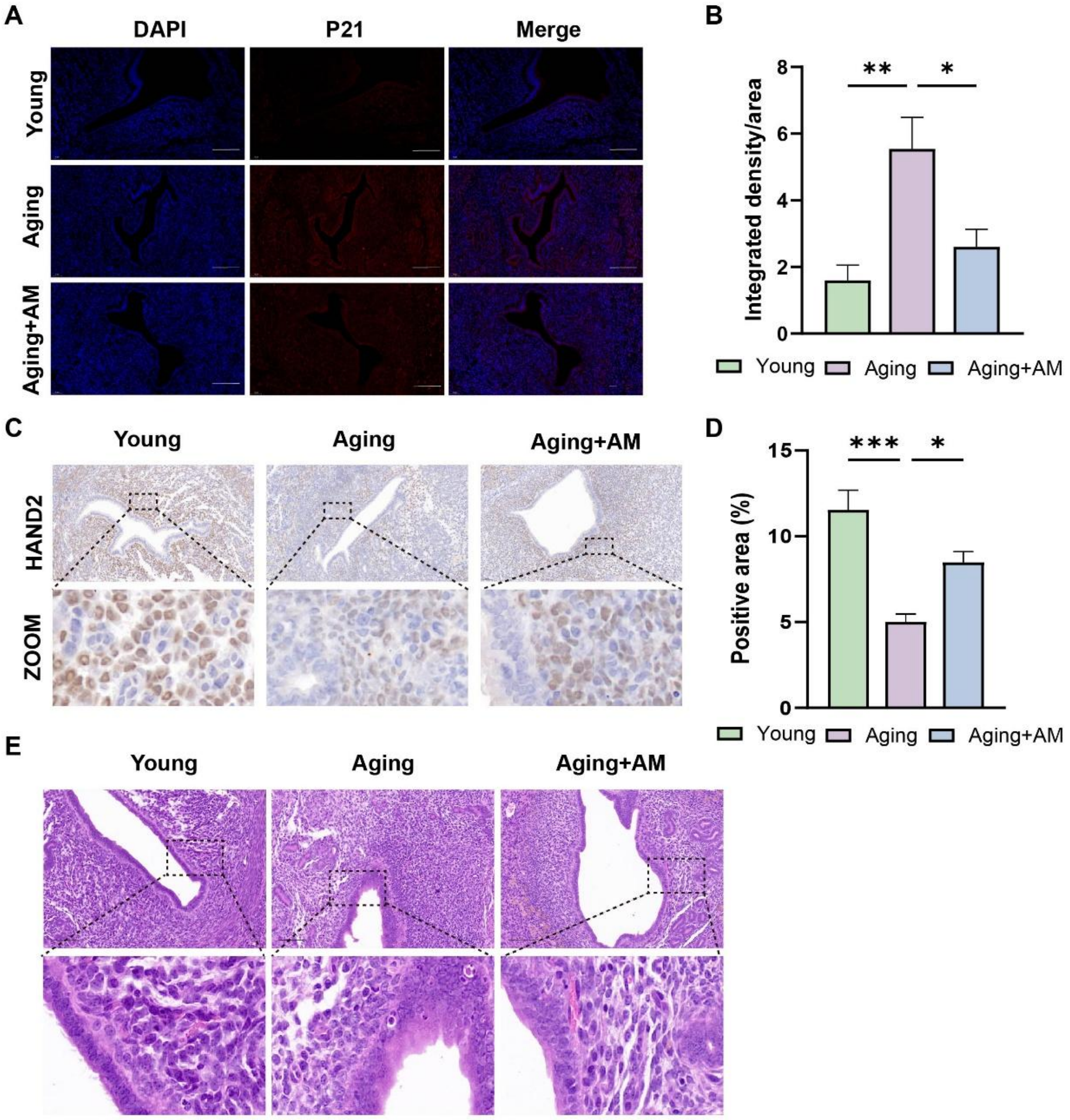
A
DAPI
P21
Merge
Young
Aging
Aging+AM
B
Integrated density/area
8
6
4
2
0
**
*
Young
Aging
Aging+AM
C
Young
Aging
Aging+AM
HAND2
ZOOM
D
Positive area (%)
15
10
5
0
***
*
Young
Aging
Aging+AM
E
Young
Aging
Aging+AM

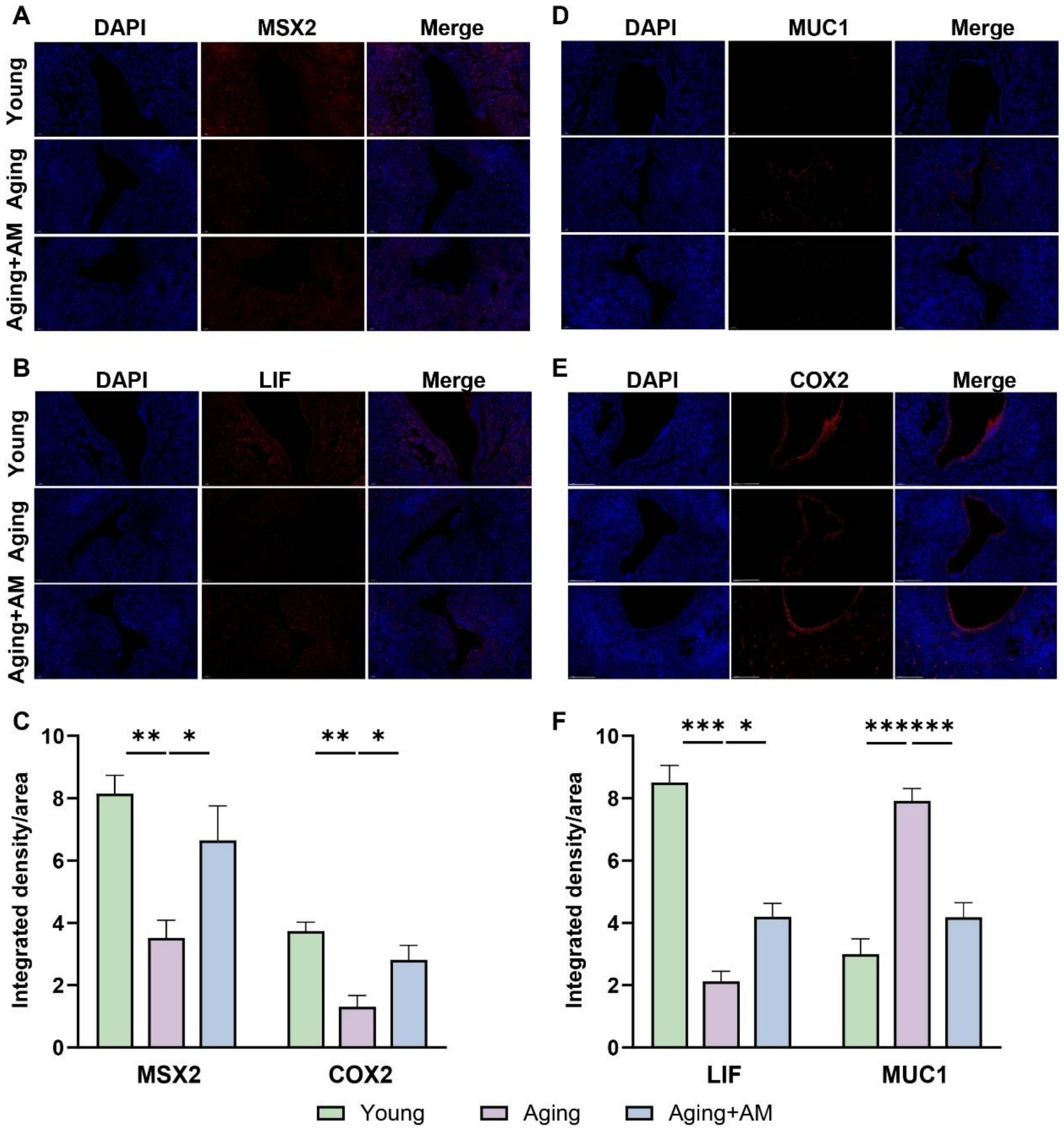
A
DAPI
MSX2
Merge
Young
Aging
Aging+AM
D
DAPI
MUC1
Merge
B
DAPI
LIF
Merge
E
DAPI
COX2
Merge
C
Integrated density/area
MSX2
COX2
F
LIF
MUC1
Young
Aging
Aging+AM

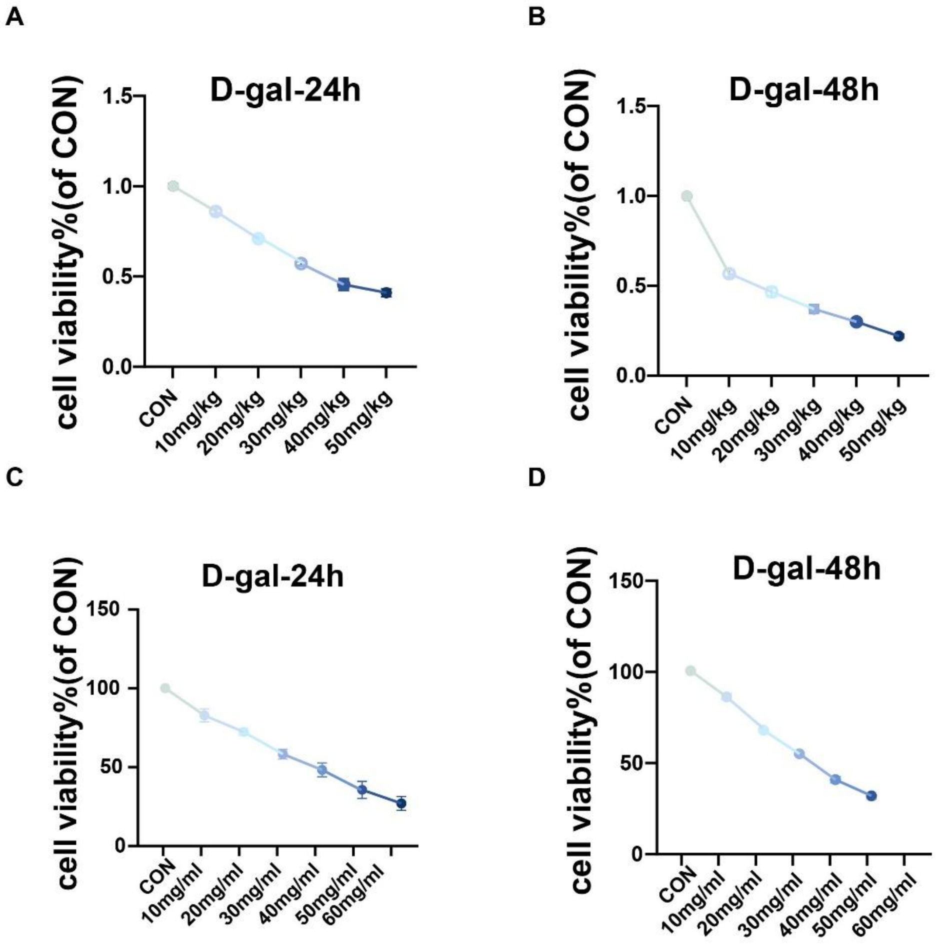
A
B
C
D
D-gal-24h
D-gal-48h
D-gal-24h
D-gal-48h
cell viability%(of CON)
1.5
1.0
0.5
0.0
150
100
50
0
CON
10mg/kg
20mg/kg
30mg/kg
40mg/kg
50mg/kg
10mg/ml
20mg/ml
30mg/ml
40mg/ml
50mg/ml
60mg/ml

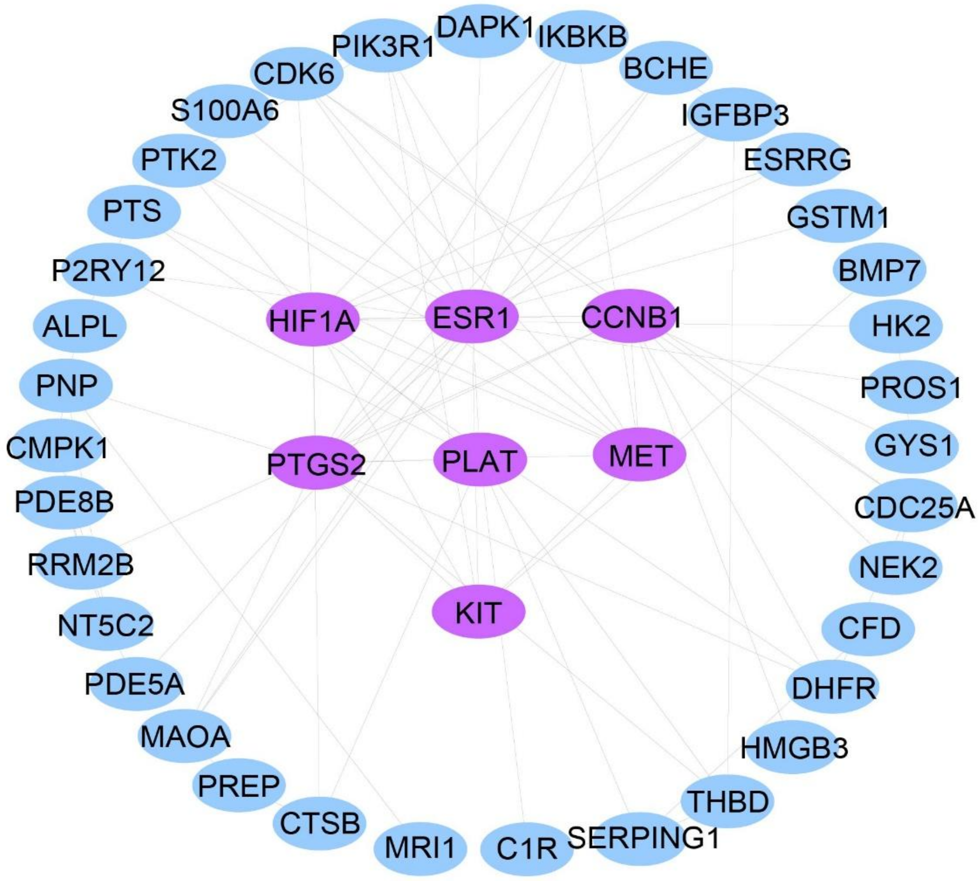

II: Supplementary Figure legends

Supplementary Figure S1. Safety evaluation of AM in aged mice. (A) Schematic illustration of the experimental design showing daily oral gavage of AM for 30 consecutive days, followed by body weight measurement and sample collection for histological and biochemical analyses. (B) Quantification of body weight among the three groups, showing no significant differences during the treatment period. (C) Representative H&E staining images of major organs, including heart, liver, spleen, lung, and kidney, from Young, Aging, and Aging + AM groups, showing no obvious pathological alterations after AM treatment (Scale bar, 50 μm). (D-G) Serum biochemical analysis of liver and kidney function indicators, including ALT, AST, CREA, and UREA, showing no significant changes among groups. Data are presented as mean ± SEM. Statistical significance was determined by one-way ANOVA: ns, not significant. (AM, *Astragalus membranaceus*; H&E, hematoxylin and eosin; ALT, alanine aminotransferase; AST, aspartate aminotransferase; CREA, creatinine; UREA, urea; Young, young mouse group; Aging, aged mouse group; Aging + AM, aged mouse group treated with *Astragalus membranaceus*)

Supplementary Figure S2. AM alleviates aging-associated phenotypes and

senescence marker expression in endometrial cells. (A) Representative SA-β-gal staining of uterine tissues from Young, Aging and Aging + AM mice. Enlarged insets show senescence-associated β-galactosidase-positive cells (Scale bar, 50 μm). (B) Relative mRNA expression levels of senescence-associated genes *CDKN2A, CDKN1A,* and *TP53* in uterine tissues measured by qRT-PCR. (C) Immunohistochemical staining of P21 in the endometrium of Young, Aging and Aging + AM mice. Boxed areas denote higher-magnification views (Scale bar, 50 μm). (D) Immunohistochemical staining of P53 in uterine tissues (Scale bar, 50 μm). (E)(F) Quantification of P21-(E) and P53-positive(F) areas based on immunohistochemistry. Data are presented as mean ± SEM. Statistical significance was determined by one-way ANOVA: $*P < 0.05$; $**P < 0.01$; $***P < 0.001$. (AM, *Astragalus membranaceus*; SA-β-gal, senescence-associated β-galactosidase; qRT -PCR, quantitative real-time polymerase chain reaction; D-gal, D-galactose; Young, young mouse group; Aging, aged mouse group; Aging + AM, aged mouse group treated with *Astragalus membranaceus*.)

Supplementary Figure S3. AM restores endometrial structural integrity and reduces nuclear senescence signals. (A) Representative immunofluorescence staining of P21 (red) with DAPI nuclear counterstaining (blue) in the endometrium. Merged images are shown (Scale bar, 50 μm). (B) Quantification of P21 fluorescence signal intensity in the indicated groups. (C) Immunohistochemical staining of Hand2 in the endometrium of Young, Aging and Aging + AM mice. Boxed areas denote higher-magnification views (Scale bar, 50 μm). (D) Quantification of Hand2-positive areas based on immunohistochemistry. (E) Representative H&E staining showing histological changes and structural organization of the endometrium in Young, Aging and Aging + AM mice (Scale bar, 50 μm). Data are presented as mean ± SEM. Statistical significance was determined by one-way ANOVA: $*P < 0.05$; $**P < 0.01$; $***P < 0.001$. (AM, *Astragalus membranaceus*; H&E, hematoxylin and eosin; DAPI, 4′,6-diamidino-2-phenylindole; Young, young mouse group; Aging, aged mouse group; Aging + AM, aged mouse group treated with *Astragalus membranaceus*.)

Supplementary Figure S4. AM restores the expression of endometrial receptivity and function-associated markers. (A, B, D, E) Representative immunofluorescence staining of MSX2 (A), LIF (B), MUC1 (D), and COX2 (E) (red) with DAPI nuclear counterstaining (blue) in the endometrium of Young, Aging, and Aging + AM mice. (Scale bar, 50 μm). (C, F) Quantitative analysis of the fluorescence intensity of MSX2 and COX2 (C), as well as LIF and MUC1 (F). Data are presented as mean ± SEM. Statistical significance was determined by one-way ANOVA: *P < 0.05; **P < 0.01; ***P < 0.001. (AM, Astragalus membranaceus; MSX2, Msh homeobox 2; LIF, Leukemia inhibitory factor; MUC1, Mucin 1; COX2, Cyclooxygenase-2; DAPI, 4′,6-diamidino-2-phenylindole; Young, young mouse group; Aging, aged mouse group; Aging + AM, aged mouse group treated with Astragalus membranaceus.)

Supplementary Figure S5. Optimization of D-galactose concentration for inducing senescence in endometrial cells. (A) Dose-response curves of cell viability in D-gal-

treated endometrial epithelial cells at 24 h. (B) Dose-response curves of cell viability in D-gal-treated endometrial epithelial cells at 48 h. (C) Dose-response curves of cell viability in D-gal-treated endometrial stromal cells at 24 h. (D) Dose-response curves of cell viability in D-gal-treated endometrial stromal cells at 48 h. Data are presented as mean ± SEM. All experiments were performed in triplicate. (D-gal, D-galactose.)

Supplementary Figure S6. PPI network analysis of the overlapping targets between AM and endometrial senescence. The PPI network shows the interactions among the core targets, with hub genes highlighted in purple (PPI, protein-protein interaction; AM, *Astragalus membranaceus*).

Supplementary Table 1. Chemical information of active compounds from AM.

Code	Name	MolID	OB	DL
**C01**	Mairin	MOL000211	55.38	0.78
**C02**	Jaranol	MOL000239	50.83	0.29
**C03**	Hederagenin	MOL000296	36.91	0.75
**C04**	(3S,8S,9S,10R,13R,14S,17R)-10,13- dimethyl -17-[(2R,5S)- 5- propan- 2-yloctan-2-yl] - 2,3,4,7,8,9,11,12,14,15,16,17-dodecahydro- 1H - cyclopenta[a]phenanthren -3- ol	MOL000033	36.23	0.78
**C05**	Isorhamnetin	MOL000354	49.60	0.31
**C06**	3,9 - di-O-methylnissolin	MOL000371	53.74	0.48
**C07**	5'-hydroxyiso- muronulatol-2',5'-di-O-glucoside	MOL000374	41.72	0.69
**C08**	7-O-methylisomucronulatol	MOL000378	74.69	0.30
**C09**	9,10 - dimethoxypterocarpan - 3-O-β-D-glucoside	MOL000379	36.74	0.92
**C10**	(6aR,11aR)-9,10- dimethoxy-6a,11a-dihydro- 6H -benzofurano[3,2-c]chromen -3-ol	MOL000380	64.26	0.42
**C11**	Bifendate	MOL000387	31.10	0.67
**C12**	Formononetin	MOL000392	69.67	0.21
**C13**	Calycosin	MOL000417	47.75	0.24
**C14**	Kaempferol	MOL000422	41.88	0.24
**C15**	(3R)-3-(2-hydroxy -3,4 - dimethoxyphenyl)chroman -7-ol	MOL000438	67.67	0.26
**C16**	Isomucronulatol -7,2'-di -O-glucosiole	MOL000439	49.28	0.62
**C17**	1,7-Dihydroxy -3,9 -dimethoxypterocarpene	MOL000442	39.05	0.48
**C18**	Quercetin	MOL000098	46.43	0.28
**C19**	Chrysanthemaxanthin	MOL004492	38.72	0.58
**C20**	Gamma- Sitosterol	MOL002320	36.91	0.75
**C21**	Folic Acid	MOL000433	68.96	0.71
**C22**	Medicarpin	MOL002565	49.22	0.34

Supplementary Table 2. Primers for qRT-PCR

(A) Mouse genes

Gene Symbol	Forward Primer Sequence (5’ to 3’)	Reverse Primer Sequence (5’ to 3’)
Cdkn2a	GCTCAACTACGGTGCAGATTC	GCACGATGTCTTGATGTCCC
Cdkn1a	CGAGAACGGTGGAACTTTGAC	CCAGGGCTCAGGTAGACCTT
Trp53	CCCCTGTCATCTTTTGTCCCT	AGCTGGCAGAATAGCTTATTGAG
Hand2	ATCGCCTACCTCATGGATCTGC	CTGCTCACTGTGCTTTTCAAGATC
Hoxa10	CTGTCTCCAGCCCCTTCAGAAA	TCTGGTGCTTCGTGTAAGGGCA
Pgr	TGTAGACCATGTAGTTGAGGTCA	CTGGCTTTGACTCCTCAGTCCT
Ltf	GTCTGCCATTGGCTTTGTGAGG	CCTTTGAGGCTATCACATCCTGC
Areg	GCAGATACATCGAGAACCTGGAG	CCTTGTCATCCTCGCTGTGAGT
Esr1	TCTGCCAAGGAGACTCGCTACT	GGTGCATTGGTTTGTAGCTGGAC
Gapdh	AGGTCGGTGTGAACGGATTTG	TGTAGACCATGTAGTTGAGGTCA

(B)Human genes

Gene Symbol	Forward Primer Sequence (5’ to 3’)	Reverse Primer Sequence (5’ to 3’)
CDKN2A	GACCTGGCTGAGGAGCTG	CAATCGGGGATGTCTGAGGG
CDKN1A	TGTCCGTCAGAACCCATGC	AAAGTCGAAGTTCCATCGCTC
TP53	CAGCACATGACGGAGGTTGT	TCATCCAAATACTCCACACGC
GAPDH	GGAGCGAGATCCCTCCAAAAT	GGCTGTTGTCATACTTCTCATGG
