# Supplementary material for: Astragalus membranaceus ameliorates endometrial aging and restores receptivity by enhancing mitochondrial function in stromal and epithelial cells
Source: Protein Cell. 2026 Jul 15;17(9):876–82. doi: 10.1093/procel/pwag012 (PMC13521270; doi:10.1093/procel/pwag012)

Figure1

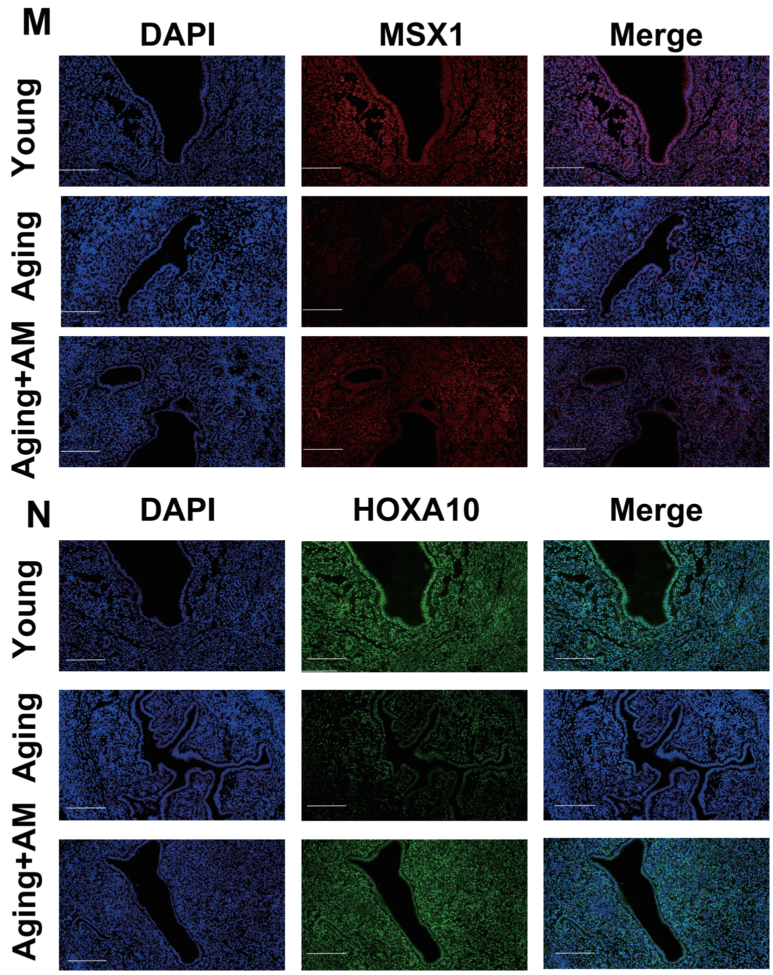

Figure 2

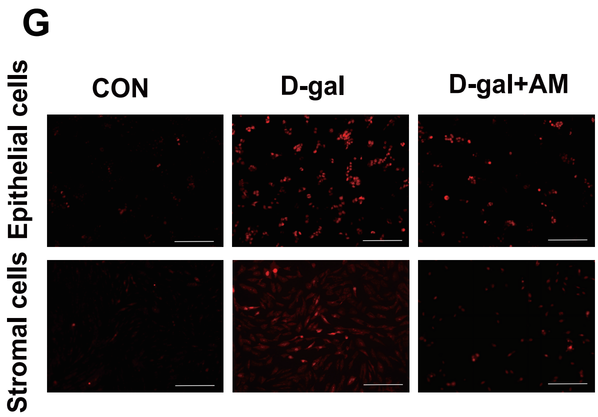

Supplement: Supplementary file 1 [file pwag012_supplementary_data.zip › pwag012_Supplementary_Data/PAC-25869_Replacement_Figures_Fig1M-1N_Fig2G.pdf]
